# Supplementary material for: Entropy-based metrics for predicting choice behavior based on local response to reward
Source: Nat Commun. 2021 Nov 12;12:6567. doi: 10.1038/s41467-021-26784-w (PMC8590026; doi:10.1038/s41467-021-26784-w)
Supplement: Supplementary file 1 — Supplementary Information [file 41467_2021_26784_MOESM1_ESM.pdf]

Supplementary Information

Entropy-based metrics for predicting choice behavior based on local response to reward

Ethan Trepka*, Mehran Spitmaan*, Bilal A. Bari, Vincent D. Costa, Jeremiah Y. Cohen, Alireza Soltani

Supplementary Note 1. Stepwise Regression for Predicting Deviation from Matching.
Four stepwise regressions were performed for each species to predict deviation from matching using behavioral and entropy-based metrics. Overall, we found that entropy-based metrics capture a substantial amount of variance in undermatching behavior and that the variance they capture goes above and beyond what can be captured by existing behavioral metrics. The order in which predictors were added to the stepwise regression models and the resulting regression equations are presented below.

Model without entropy-based metrics or repetition indices

Mice: In the first step of the stepwise regression, $p(win)$ entered the regression equation as a significant predictor of undermatching, $RMSE = 0.1119, p = 4.12 \times 10^{-175}$. In the next steps, $p(stay)$ ($RMSE = 0.1053, p = 2.41 \times 10^{-89}$) and $p(stay|win)$ ($RMSE = 0.1046, p = 7.41 \times 10^{-11}$) entered the regression equation. This resulted in the following equation for predicting deviation from matching:

$$Dev. from\ matching_{Mice} = 0.67 \times p(win) + 0.19 \times p(stay) + 0.12 \times p(stay|win) - 0.65 \quad (1)$$

Monkeys: In the first step of the stepwise regression, $p(stay)$ entered the regression equation as a significant predictor of undermatching, $RMSE = 0.0599, p = 1.31 \times 10^{-160}$. In the next steps, $p(win)$ ($RMSE = 0.0579, p = 1.86 \times 10^{-35}$), $p(switch|lose)$ ($RMSE = 0.0575, p = 2.85 \times 10^{-8}$), and $p(stay|win)$ ($RMSE = 0.0571, p = 5.61 \times 10^{-9}$) entered the regression equation. In the final step, $p(stay)$ was removed from the equation because it was no longer a significant predictor ($RMSE = 0.0572, p = 6.37 \times 10^{-4}$). This resulted in the following equation for predicting deviation from matching:

$$Dev. from\ matching_{Monkeys} = 0.23 \times p(win) - 0.12 \times p(switch|lose) + 0.12 \times p(stay|win) - 0.33. \quad (2)$$

In the final regression model, $p(win)$ and $p(stay|win)$ all had positive regression coefficients for both mice and monkeys, indicating that increased $p(win)$ and $p(stay|win)$ are associated with less undermatching behavior (note that deviation from matching is mostly negative).

Model without entropy-based metrics

Mice: In the first step of the regression process, $p(win)$ entered the model ($RMSE = 0.1119, p < 4.12 \times 10^{-175}$). In the next steps, $p(stay)(RMSE = 0.1053, p < 2.41 \times 10^{-89})$, RI_W $(RMSE = 0.0929, p < 4.80 \times 10^{-181})$, $p(stay|win)(RMSE = 0.0914, p < 1.03 \times 10^{-24})$, and $RI_B(RMSE = 0.0910, p < 2.98 \times 10^{-8})$ were added to the regression equation. The final regression equations for predicting deviation from matching for mice and monkeys was:

$$Dev.from\ matching_{Mice} = 0.42 \times p(win) + 0.45 \times p(stay) + 0.17 \times p(stay|win) - 0.44 \times RI_B - 0.75 \times RI_W - 0.73. \quad (3)$$

Monkeys: In the first step of the regression process, $p(stay)$ entered the model ($RMSE = 0.0599, p = 1.31 \times 10^{-160}$). In the next steps, $RI_B(RMSE = 0.0505, p = 6.48 \times 10^{-167})$ and $p(switch|lose)(RMSE = 0.0503, p < 8.59 \times 10^{-5})$ were added to the regression equation. The final regression equations for predicting deviation from matching for monkeys was:

$$Dev.from\ matching_{Monkeys} = 0.32 \times p(stay) - 0.57 \times RI_B - 0.04 \times p(switch|lose) - 0.33. \quad (4)$$

RI_B and RI_W both had negative coefficients in the regression equations they were present in, indicating that repeating better or worse option beyond chance increases undermatching. This was expected for RI_W because staying beyond chance on the worse option (worse side or stimulus) results in more frequent selection of the worse option and thus more undermatching. In contrast, larger RI_B could increase undermatching because more staying on the better option could stop the animals from switching to the new better option after block switches.

Full model

Mice: In the first step of the regression process, $ERODS_{W-}$ entered the regression equation as a significant predictor of deviation from matching, $RMSE = 0.0717, p < 10^{-300}$. Next, $ERODS_{W+}$ entered the regression equation, $RMSE = 0.0681, p < 1.61 \times 10^{-65}$. In the

following steps, $ERODS_{B+}$ ($RMSE = 0.0666, p < 1.97 \times 10^{-29}$), $ERDS_{+}$ ($RMSE = 0.0658, p < 4.11 \times 10^{-18}$), $EODS_{W}$ ($RMSE = 0.0651, p < 1.72 \times 10^{-15}$), $P(switch|lose)$ ($RMSE = 0.0639, p < 7.54 \times 10^{-24}$), RI_{W}($RMSE = 0.0617, p < 1.46 \times 10^{-46}$), $P(stay|win)$($RMSE = 0.0585, p < 1.81 \times 10^{-67}$), $ERODS_{B-}$ ($RMSE = 0.0573, p < 5.15 \times 10^{-28}$), $ERDS_{-}$ ($RMSE = 0.0568, p < 9.59 \times 10^{-13}$), $p(win)$ ($RMSE = 0.0566, p < 3.75 \times 10^{-6}$), and $EODS_{B}$($RMSE = 0.0564, p < 3.28 \times 10^{-5}$) were added to the regression equation. In the final step, $ERODS_{B+}$ ($RMSE = 0.0565, p < 5.32 \times 10^{-3}$) was removed from the equation.

The final regression equation for predicting deviation from matching using all metrics in mice was as follows:

$$Dev. from\ matching_{Mice} = -0.85 \times ERODS_{W-} + 0.32 \times ERODS_{W+} - 0.22 \times ERODS_{B+} + 0.22 \times ERDS_{+} + 0.78 \times EODS_{W} - 0.37 \times P(switch|lose) - 1.09 \times RI_{W} + 0.33 \times P(stay|win) + 0.57 \times ERODS_{B-} - 0.17 \times ERDS_{-} + 0.11 \times p(win) - 0.22 \times EODS_{B} - 0.30 \quad (5)$$

Monkeys: In the first step of the regression process, $ERODS_{W-}$ entered the regression equation as a significant predictor of deviation from matching, $RMSE = 0.0589, p < 7.31 \times 10^{-114}$. Next, $EODS_{W}$ entered the regression equation, $RMSE = 0.0551, p < 1.50 \times 10^{-41}$. In the following steps, $ERODS_{B+}$ ($RMSE = 0.0525, p < 2.15 \times 10^{-31}$), RI_{B} ($RMSE = 0.0512, p < 1.09 \times 10^{-16}$), $P(switch|lose)$($RMSE = 0.0477, p < 1.10 \times 10^{-43}$), $P(stay|win)$ ($RMSE = 0.0468, p < 2.02 \times 10^{-13}$), and $ERDS_{+}$($RMSE = 0.0463, p < 5.44 \times 10^{-8}$) were added to the regression equation.

The final regression equation for predicting deviation from matching using all metrics in monkeys was as follows:

$$Dev. from\ matching_{Monkeys} = -1.02 \times ERODS_{W-} + 0.16 \times ERDS_{+} + 0.33 \times P(stay|win) - 0.21 \times P(switch|lose) - 0.66 \times RI_{B} - 0.23 \times ERODS_{B+} + 0.83 \times EODS_{W} - 0.31. \quad (6)$$

The coefficients of predictors in this model cannot be interpreted in isolation in this model due to multicollinearity among entropy-based metrics.

Given the complexity of the final equation to predict deviation from matching, we also constructed simpler linear regression models predicting deviation from matching using the first three entropy-based metrics added to the stepwise regressions ($ERODS_{W-}$, $ERODS_{W+}, and\ ERODS_{B+}$ for mice, and $ERODS_{W-},\ ERODS_{B+}, and\ EODS_{W}$ for monkeys). For mice, the regression equation for this simple model was: $Dev. from\ matching = -0.62 \times ERODS_{W-} + 0.73 \times ERODS_{W+} - 0.16 \times ERODS_{B+} - 0.02$. For monkeys, the regression equation for this simple model was: $Dev. from\ matching = -1.09 \times ERODS_{W-} - 0.14 \times ERODS_{B+} + 0.63 \times EODS_{W} - 0.06$. Despite these models' simplicity, they still explained 64% of total variance in deviation from matching for mice and 45% for monkeys (Monkeys: $Adjusted\ R^2 = 0.45$; Mice: $Adjusted\ R^2 = 0.64$).

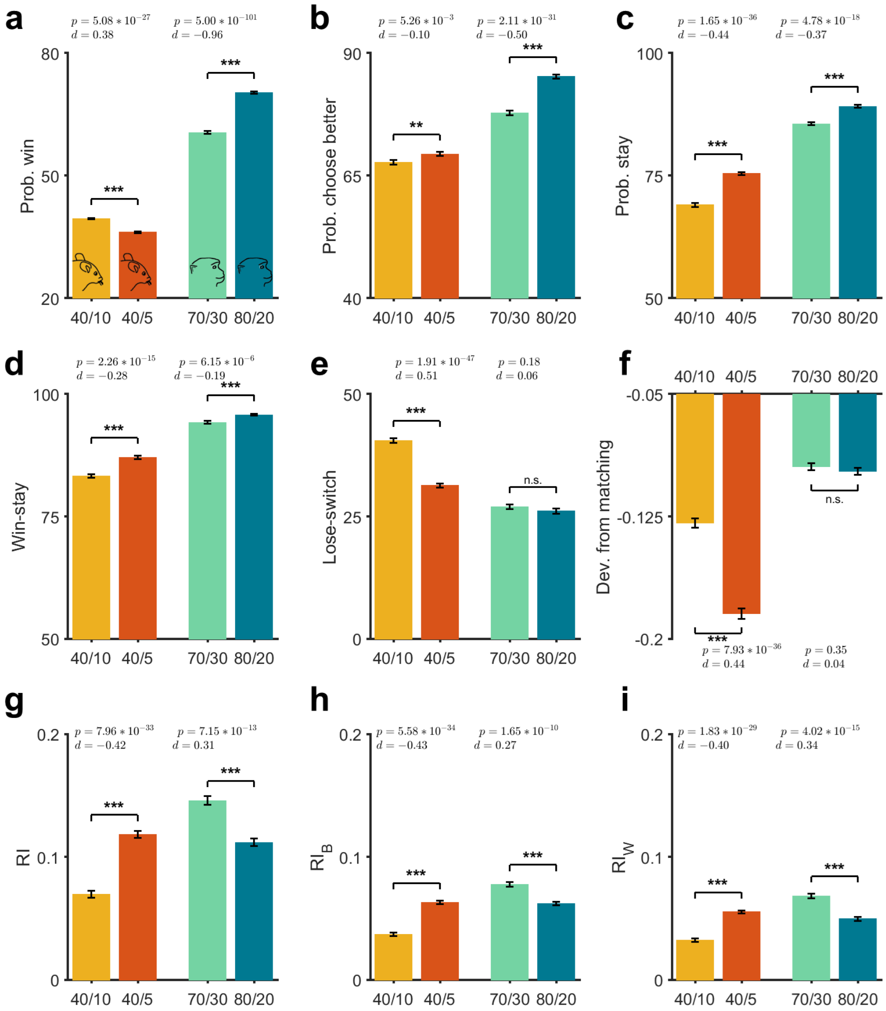

Supplementary Figure 1. Undermatching and behavioral metrics depend on reward probabilities. Plotted are the probability of winning (a), the probability of choosing the better option (b), probability of staying on previous choice (c), win-stay (d), lose-switch (e), deviation from matching (f), RI (i), and RI for the better (h) and worse options (i), separately in the 40/10 and 40/5 reward schedules for mice and the 70/30 and 80/20 reward schedules for monkeys. Error bars indicate s.e.m. For mice, $n_{40/5} = 1786$ blocks and $n_{40/10} = 1533$ blocks, and for monkeys, $n_{70/30} = 1110$ blocks and $n_{80/20} = 1102$ blocks. The asterisk indicates a significant difference between the two environments using two-sided t-test with p-value and Cohen's d-value reported on each panel. In mice, the probability of winning was significantly higher in the 40/10 schedule despite a lower probability of choosing the better side. In monkeys, the probability of winning and probability of choosing better was higher in the 80/20 schedule. Moreover, the probability of staying and the repetition index were both significantly lower in the 40/10 schedule in mice and 70/30 schedule in monkeys because the reward probabilities for the two options are more similar. Finally, both win-stay and lose-switch were closer to 0.5 in the 40/10 schedule for mice, and win-stay was closer to 0.5 in the 70/30 schedule for monkeys which may indicate a decrease in the dependence of staying and switching behavior on reward. However, the differences in p(win) and p(stay) between these environments make interpreting win-stay and lose-switch in isolation challenging.

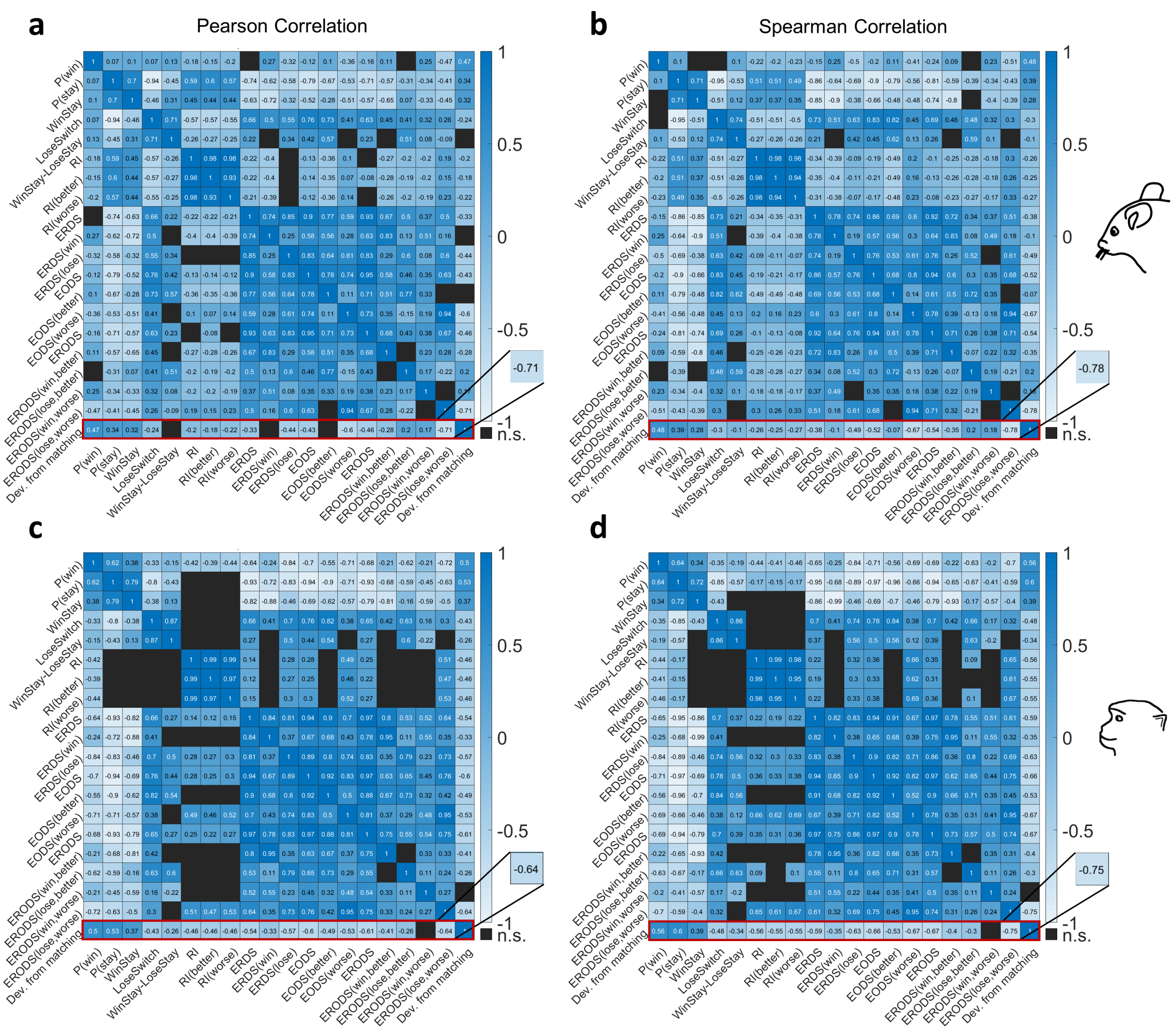

Supplementary Figure 2. Correlation between undermatching and proposed entropy-based metrics and underlying probabilities. (a–b) Correlation matrix for 19 behavioral metrics and undermatching in mice using Pearson (a) and Spearman (b) tests. Correlation coefficients are computed across all blocks, and matrix elements with non-significant values (two-sided, $p >$ .0001) are not shown (cells in black). The red rectangles highlight correlation coefficients between behavioral metrics and undermatching. **(c–d)** Similar to (a–b) but for monkeys. Overall, the entropy-based metrics show stronger correlation with undermatching than previous metrics, and undermatching was most strongly correlated with ERODS$_{W-}$, EODS$_{W}$, and ERDS$_{-}$.

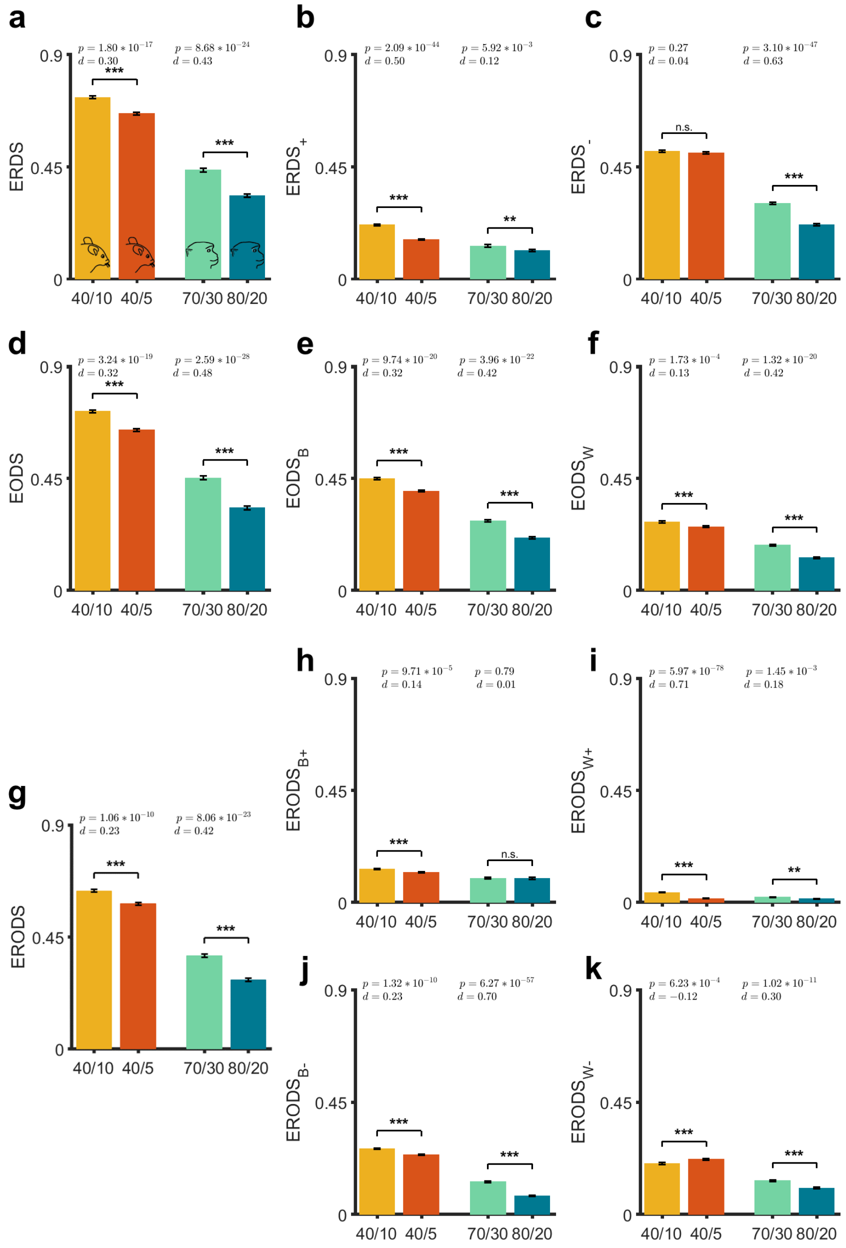

Supplementary Figure 3. Entropy-based metrics capture changes in learning strategy between the two reward schedules. Plotted are ERDS (a) and ERDS decompositions (b–c), EODS (d) and EODS decompositions (e–f), and ERODS (g) and ERODS decompositions (h–k), separately in the 40/10 and 40/5 reward schedules for mice and the 70/30 and 80/20 reward schedules for monkeys. Error bars indicate s.e.m. For mice, $n_{40/5} = 1786$ blocks and $n_{40/10} = 1533$ blocks, and for monkeys, $n_{70/30} = 1110$ blocks and $n_{80/20} = 1102$ blocks. Reported are the p-values (two-sided t-test) and Cohen's d-values. ERDS, EODS, and ERODS were significantly higher in the 40/10 schedule in mice and the 70/30 schedule in monkeys. Overall, increased entropy in the 40/10 schedule in mice and the 70/30 schedule in monkeys suggests a decrease in the consistency of reward and option-dependent strategy. Decreased consistency of reward and option-dependent strategy may be due to the greater similarity of reward probabilities for the two options in the 40/10 and 70/30 reward schedules.

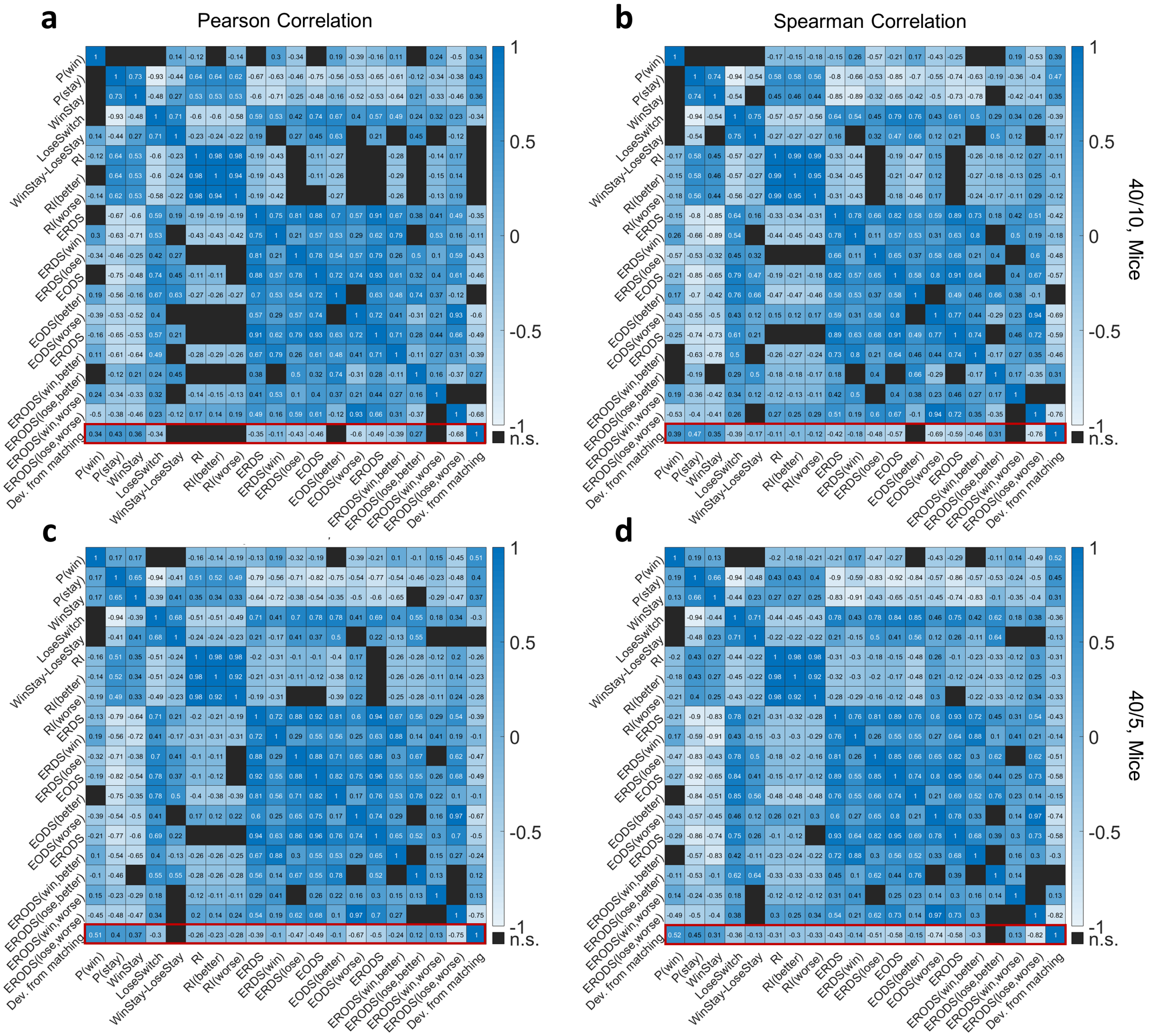

Supplementary Figure 4. Correlation between undermatching and proposed entropy-based metrics separately for each reward schedule in mice. Correlation matrix for 19 behavioral metrics and undermatching using Pearson (a) and Spearman (b) tests, separately for blocks with reward probabilities equal to 40/10 (a–b) or 40/5 (c–d). Correlation coefficients are computed across all blocks, and matrix elements with non-significant values (two-sided, $p > .0001$) are not shown (cells in black). The red rectangles highlight correlation coefficients between behavioral metrics and undermatching. Overall, the entropy-based metrics were similarly strongly correlated with undermatching in both reward environments.

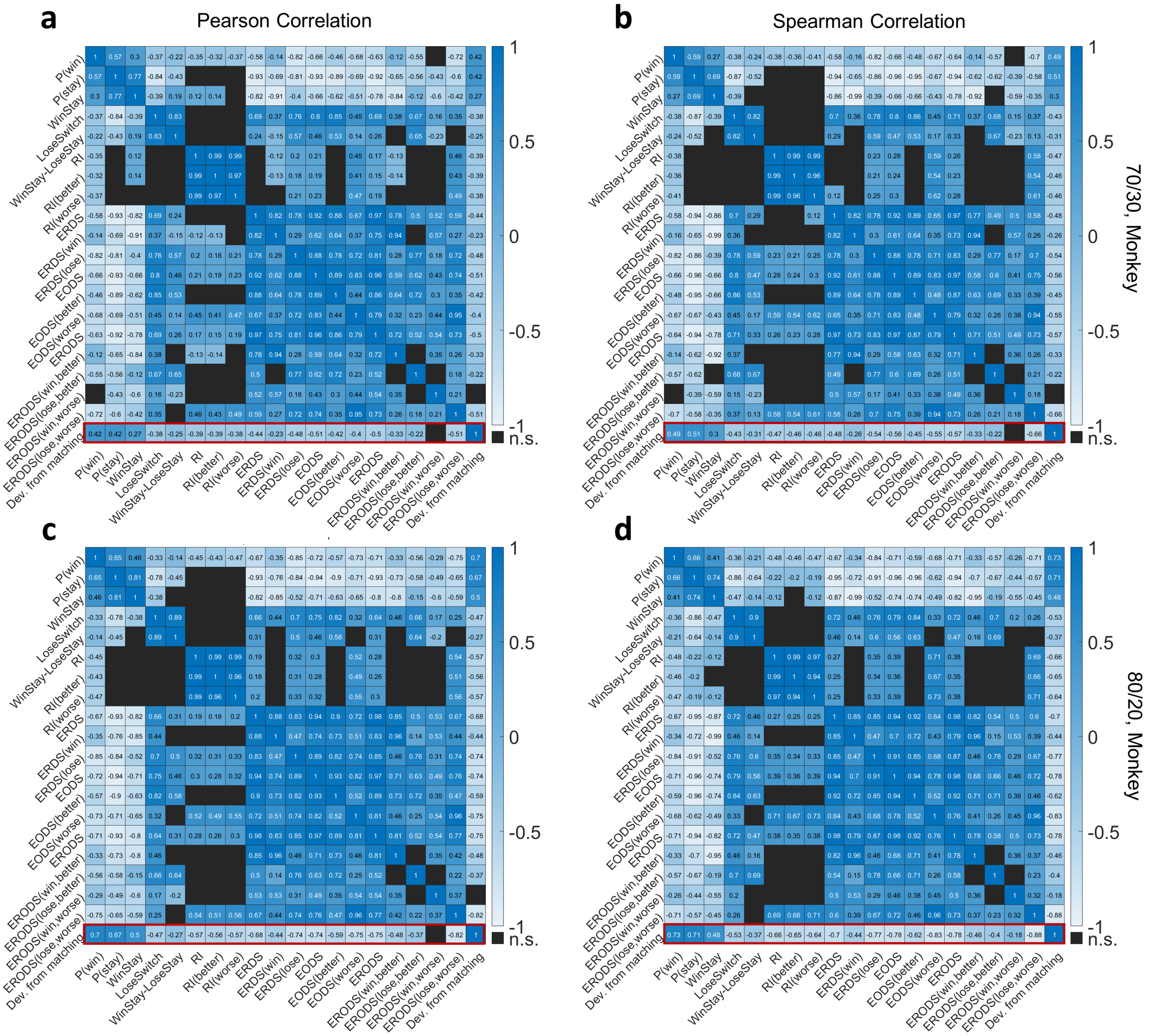

Supplementary Figure 5. Correlation between undermatching and proposed entropy-based metrics separately for each reward schedule in monkeys. Same as Supplementary Figure 4 but for monkeys, separately for blocks with reward probabilities equal to 70/30 (a–b) or 80/10 (c–d). Correlation coefficients are computed across all blocks, and matrix elements with non-significant values (two-sided, $p > .0001$) are not shown (cells in black). Overall, the entropy-based metrics were similarly strongly correlated with undermatching in both reward environments.

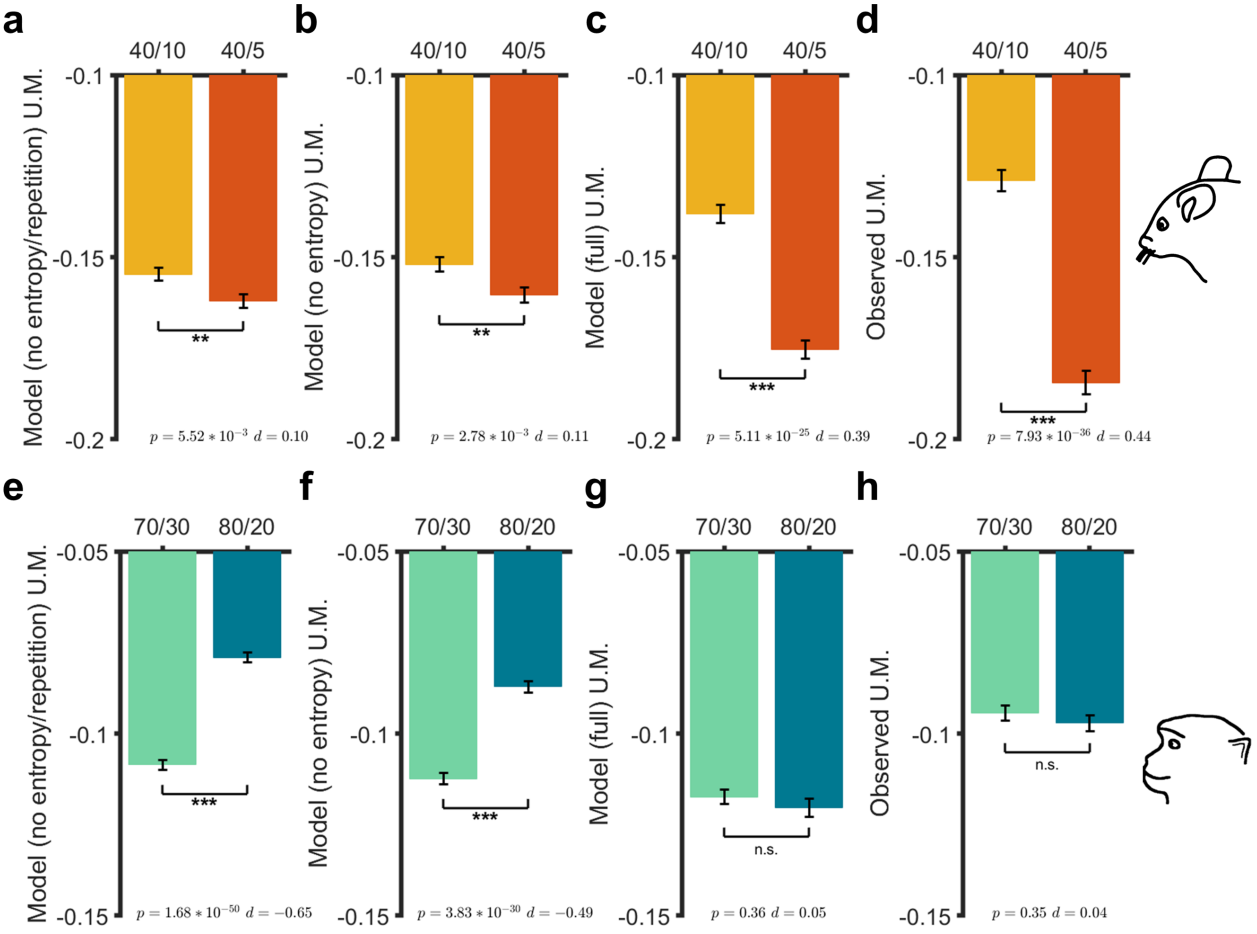

Supplementary Figure 6. Entropy-based metrics capture differences in undermatching between reward environments. (a–d) Plotted are deviation from matching (undermatching, U.M.) in the 40/10 and 40/5 reward schedules for mice predicted from 10-fold cross-validated linear regression models using all behavioral metrics except entropy and repetition metrics as predictors (a), all behavioral metrics except entropy-based metrics as predictors (b), and all metrics as predictors (c) versus observed deviation from matching (d). Predictors were selected for inclusion using the stepwise regressions described in the manuscript, then 10-fold cross-validated linear regression was performed to fit models and predict undermatching (see **Methods**). Error bars indicate s.e.m. Reported are the p-values (two-sided t-test) on the left and Cohen's d-values on the right. No adjustments were made for multiple comparisons. **(e–h)** Similar to (a–d), but with monkey data in the 70/30 and 80/20 reward schedules. Error bars indicate s.e.m. Reported are the p-values (two-sided t-test) on the left and Cohen's d-values on the right. No adjustments were made for multiple comparisons. For mice (a-d), $n_{40/5} =$ 1786 blocks and $n_{40/10} = 1533$ blocks, and for monkeys (e-d), $n_{70/30} =$ 1110 blocks and $n_{80/20} = 1102$ blocks. The full regression model replicates the observed differences between reward schedules in mice and the lack of observed differences between reward schedules in monkeys.

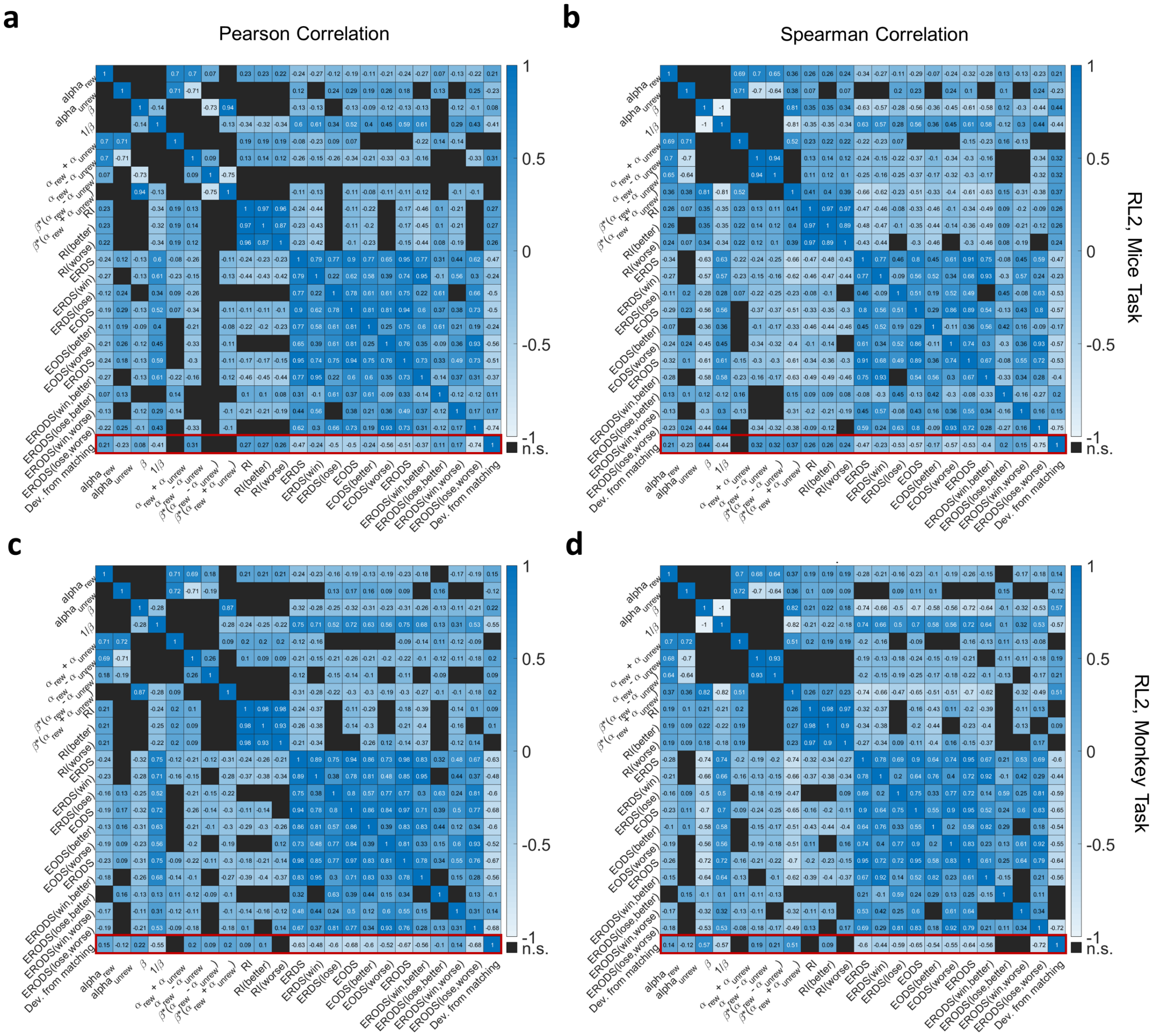

Supplementary Figure 7. **Undermatching in the RL model was better predicted by entropy-based metrics than parameters of the RL model.** Plotted are correlation matrices for entropy-based metrics and deviation from matching computed over generated choice from block-wise simulations of RL2 with random parameters, separately based on Pearson (a,c) and Spearman (b,d) tests. Simulations were done using both the mice task setup and monkey task setup (a–b and c–d, respectively). Correlation coefficients are computed across all blocks, and matrix elements with non-significant values (two-sided, $p < .0001$) are not shown (cells in black). The red rectangles highlight correlation coefficients between metrics and undermatching. Temperature, $1/\beta$, that measures sensitivity of choice to value differences was the best correlate of deviation from matching out of all the RL parameters for both animals (Pearson: mice: $r =$ -0.41, monkeys: $r =$ -0.55; Spearman: mice: $r =$ -0.44, monkeys: $r =$ -0.57). In contrast, ERODS_{W-} was the best correlate of deviation from matching out of all entropy-based metrics (Pearson: mice: $r =$ -0.74, monkeys: $r =$ -0.68; Spearman: mice: $r =$ -0.75, monkeys: $r =$ -0.72). Interestingly, entropy-based metrics were also highly correlated with $1/\beta$, suggesting that they capture explore/exploit dynamics (see columns 3–4 of matrices). Overall, the entropy-based metrics predict deviation from matching better than the parameters of the RL models.

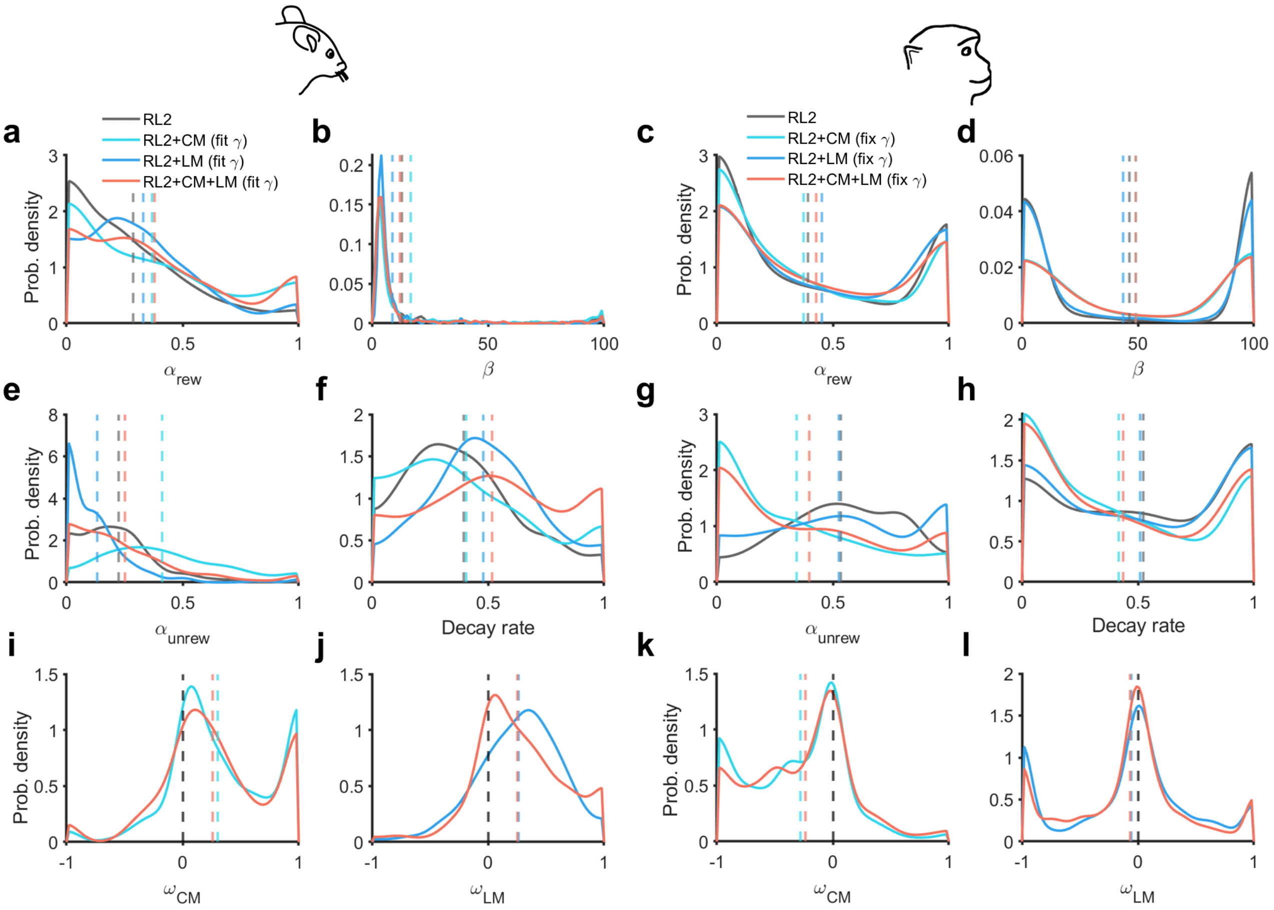

Supplementary Figure 8. **Distributions of model parameters obtained from fitting choice behavior.** Plotted are distributions of the learning rate on the rewarded option (a,c), inverse temperature (β) measuring sensitivity to value differences (b,d), learning rate on the unrewarded action/option (e,g), decay rate (f,h), the weight of the choice memory component (i,j), and the weight of the lose-memory component (k,l) for mice (a-j) and monkeys (c-l) using four reinforcement learning models as noted in the legend. Probability density curves are estimated using kernel smoothing with reflection for boundary correction. Dashed vertical lines indicate the mean of each distribution in all plots and the black dashed vertical lines in (i-l) are zero lines. In mice, the choice and loss memory components have positive weights, whereas in monkeys the choice and loss memory components have slightly negative weights.

Metric	Decomposition	Description	Components
**ERDS**		Entropy of **reward-**dependent strategy.	$p(win)$, $p(stay\|win)$, $p(switch\|lose)$
	**ERDS**$_{+}$	Entropy of **win-**dependent strategy.	$p(win)$, $p(stay\|win)$
	**ERDS**$_{-}$	Entropy of **loss-**dependent strategy	$p(lose)$, $p(switch\|lose)$
**EODS**		Entropy of **option**-dependent strategy	$p(choose\ better)$, $p(stay\|choose\ better)$, $p(switch\|choose\ worse$
	**EODS**$_{B}$	Entropy of **option**-dependent strategy on the **better side**.	$p(choose\ better)$, $p(stay\|choose\ better)$
	**EODS**$_{W}$	Entropy of **option**-dependent strategy on the **worse side**.	$p(choose\ worse)$, $p(switch\|choose\ worse$
**ERODS**		Entropy of **reward- and option-** dependent strategy	$p(win, better)$, $p(win, worse)$, $p(lose, better)$, $p(lose, worse)$, $p(stay\|win, better)$, $p(stay\|win, worse)$, $p(switch\|lose, better)$, $p(switch\|lose, worse)$
	**ERODS**$_{B+}$	Entropy of **reward- and option-** dependent strategy in response to **win** after selecting the **better option.**	$p(win, better)$, $p(stay\|win, better)$
	**ERODS**$_{B-}$	Entropy of **reward- and option-** dependent strategy in response to **loss** after selecting the **better option.**	$p(lose, better)$, $p(switch\|lose, better)$
	**ERODS**$_{W+}$	Entropy of **reward- and option-** dependent strategy in response to **win** after selecting the **worse option.**	$p(win, worse)$, $p(stay\|win, worse)$,
	**ERODS**$_{W-}$	Entropy of **reward- and option-** dependent strategy in response to **loss** after selecting the **worse option.**	$p(lose, worse)$, $p(switch\|lose, worse)$

Supplementary Table 1. Summary and components of entropy-based metrics. Each row contains a short description of a metric, its decompositions, and the set of component probabilities that can be used to compute the metric.

Model	Model Description	Parameters	-LL	MF R^2	AIC	*D* ERODSw-	*D* matching
**RL1**	Return-based RL	α_{rew}, α_{unrew}, β 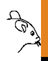	228.31	0.184	462.62 (89.65*)	0.238	0.224
		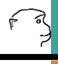	21.05	0.620	48.11 (5.01*)	0.054	0.092
**RL2**	Income-based RL	α_{rew}, α_{unrew}, β, $decay_{rate}$	189.61	0.322	387.22 (14.25*)	0.121	0.091
			18.09	0.674	44.18 (1.08*)	0.072	0.101
**Multiple Timescales**	Parallel learning on multiple timescales	ω_{fast-1}, ω_{fast-2}, ω_{slow}	198.49	0.290	402.99 (30.02*)	0.188	0.165
			21.88	0.605	49.76 (6.66*)	0.127	0.164
**RL1+CM**	Return-based RL + full choice memory	α_{rew}, α_{unrew}, β, ω_{CM},γ	190.72	0.318	391.44 (18.41*)	0.095	0.088
			18.24	0.671	46.47 (3.37*)	0.027	0.072
**RL2+ CM**	Income-based RL + choice memory	α_{rew}, α_{unrew}, β, $decay_{rate}$, ω_{CM}, γ (for mice only)	184.84	0.339	381.70 (11.00*)	0.095	0.077
			**16.54**	**0.702**	**43.10 (0)**	**0.037**	**0.065**
**RL2+ CM+**	Income-based RL + choice memory with positive CM weight	α_{rew}, α_{unrew}, β, $decay_{rate}$, ω_{CM}, γ (for mice only)	185.74	0.336	383.48 (10.51*)	0.106	0.083
			17.90	0.677	45.79 (2.69*)	0.072	0.101
**RL2+ LM**	Income-based RL + loss memory	α_{rew}, α_{unrew}, β, $decay_{rate}$, ω_{LM}, γ (for mice only)	182.24	0.348	376.47 (3.50*)	0.060	0.077
			17.06	0.692	44.13 (1.03*)	0.060	0.089
**RL2+ LM+**	Income-based RL + loss memory with positive LM weight	α_{rew}, α_{unrew}, β, $decay_{rate}$, ω_{LM}, γ (for mice only)	182.30	0.348	376.59 (3.62*)	0.059	0.078
			17.07	0.692	44.14 (1.04*)	0.060	0.089
**RL2+ CM+ LM**	Income-based RL + loss memory + choice memory	α_{rew}, α_{unrew}, β, $decay_{rate}$, ω_{CM}, ω_{LM}, γ (for mice only)	**179.49**	**0.358**	**372.97 (0)**	**0.049**	**0.065**
			15.82	0.715	43.64 (0.54*)	0.040	0.067

Supplementary Table 2. **Various models used to fit choice data, their parameters and goodness-of-fit measures, and models' ability to capture behavioral metrics.** Each row provides a short description of a given model, its parameters, goodness-of-fit based on the negative log-likelihood values separately for mice and monkeys (column 4), McFadden R^2 values or variance in choice explained by each model (column 5), goodness-of-fit based on the AIC (column 6), D-values based on Kolmogorov-Smirnov tests comparing distributions of ERODSw- (column 7) and deviation from matching (column 8) predicted using model simulations and actual behavior. Values reported in parentheses and the asterisks in column 6 indicate the difference in AIC of a given model and the full model and the significance of this difference using paired-samples t-test ($p < 1.0 \times 10^{-8}$). Rows in orange and cyan correspond to mouse and monkey data, respectively.
