# Supplementary material for: Entropy-based metrics for predicting choice behavior based on local response to reward
Source: Nat Commun. 2021 Nov 12;12:6567. doi: 10.1038/s41467-021-26784-w (PMC8590026; doi:10.1038/s41467-021-26784-w)
Supplement: Supplementary file 3 — Reporting summary [file 41467_2021_26784_MOESM3_ESM.pdf]

nature research

Corresponding author(s): Alireza Soltani

Last updated by author(s): Sep 11, 2021

Reporting Summary

Nature Research wishes to improve the reproducibility of the work that we publish. This form provides structure for consistency and transparency in reporting. For further information on Nature Research policies, see our Editorial Policies and the Editorial Policy Checklist.

Statistics

For all statistical analyses, confirm that the following items are present in the figure legend, table legend, main text, or Methods section.

n/a	Confirmed	
☐	☒	The exact sample size (n) for each experimental group/condition, given as a discrete number and unit of measurement
☒	☐	A statement on whether measurements were taken from distinct samples or whether the same sample was measured repeatedly
☐	☒	The statistical test(s) used AND whether they are one- or two-sided<br>*Only common tests should be described solely by name; describe more complex techniques in the Methods section.*
☐	☒	A description of all covariates tested
☐	☒	A description of any assumptions or corrections, such as tests of normality and adjustment for multiple comparisons
☐	☒	A full description of the statistical parameters including central tendency (e.g. means) or other basic estimates (e.g. regression coefficient) AND variation (e.g. standard deviation) or associated estimates of uncertainty (e.g. confidence intervals)
☐	☒	For null hypothesis testing, the test statistic (e.g. F, t, r) with confidence intervals, effect sizes, degrees of freedom and P value noted<br>*Give P values as exact values whenever suitable.*
☒	☐	For Bayesian analysis, information on the choice of priors and Markov chain Monte Carlo settings
☒	☐	For hierarchical and complex designs, identification of the appropriate level for tests and full reporting of outcomes
☐	☒	Estimates of effect sizes (e.g. Cohen's d, Pearson's r), indicating how they were calculated

Our web collection on statistics for biologists contains articles on many of the points above.

Software and code

Policy information about availability of computer code

Data collection	For monkey experiments, stimulus presentation and behavioral monitoring were controlled using MonkeyLogic v1.1. For mice, task events were controlled with a programmed ATmega16U2 or ATmega328 microcontroller.
Data analysis	All analysis code was written in MATLAB R2019a and is available at https://doi.org/10.5281/zenodo.5501693.

For manuscripts utilizing custom algorithms or software that are central to the research but not yet described in published literature, software must be made available to editors and reviewers. We strongly encourage code deposition in a community repository (e.g. GitHub). See the Nature Research guidelines for submitting code & software for further information.

Data

Policy information about availability of data

All manuscripts must include a data availability statement. This statement should provide the following information, where applicable:

- Accession codes, unique identifiers, or web links for publicly available datasets
- A list of figures that have associated raw data
- A description of any restrictions on data availability

The data analyzed in this study is availabe at https://doi.org/10.5281/zenodo.5501693.

Field-specific reporting

Life sciences study design

All studies must disclose on these points even when the disclosure is negative.

Sample size	No sample-size calculation was performed. Mice and monkey experiments both use large sample sizes that are sufficient for the analyses performed here. Sample size was based on what is typically used in the field due to different resource constraints associated with working with different species (>1 monkey, >10 mice).
Data exclusions	Data from the 60/40 reward schedule in monkeys was not analyzed here because it involved a level of uncertainty different from reward schedules used in mouse experiment.
Replication	Experimental findings were verified across two species, mice and monkeys, and two reward schedules for each species. Specifically, analyses were completed for mice data then subsequently replicated on monkey data. Moreover, data was divided by reward schedule and the main behavioral results were verified independently for different reward schedules as shown in the supplement.
Randomization	Randomization was not relevant to the study because animals were not assigned to control/experimental groups. All mice completed blocks of both the 5/40 and 10/40 reward schedules and all monkeys completed blocks of both the 70/30 and 80/20 reward schedules. Thus, animals did not need to be randomly assigned to different conditions because they completed all conditions. Nonetheless, the different reward schedules were randomly interleaved within a session for monkeys and across sessions for mice.
Blinding	Blinding is not relevant to the study because animals were not assigned to control/experimental groups.

Reporting for specific materials, systems and methods

We require information from authors about some types of materials, experimental systems and methods used in many studies. Here, indicate whether each material, system or method listed is relevant to your study. If you are not sure if a list item applies to your research, read the appropriate section before selecting a response.

Materials & experimental systems

n/a	Involved in the study
☒	☐ Antibodies
☒	☐ Eukaryotic cell lines
☒	☐ Palaeontology and archaeology
☐	☒ Animals and other organisms
☒	☐ Human research participants
☒	☐ Clinical data
☒	☐ Dual use research of concern

Methods

n/a	Involved in the study
☒	☐ ChIP-seq
☒	☐ Flow cytometry
☒	☐ MRI-based neuroimaging

Animals and other organisms

Policy information about studies involving animals; ARRIVE guidelines recommended for reporting animal research

Laboratory animals	Mice, C57BL/6J (The Jackson Laboratory, 000664), male, 6-20 weeks old; Monkeys, 5-6 years old, rhesus macaques, male
Wild animals	The study did not involve wild animals.
Field-collected samples	The study did not involve samples collected from the field.
Ethics oversight	The National Institute of Mental Health Animal Care and Use Committee approved the experiments in monkeys and the Johns Hopkins University Animal Care and Use Committee approved the experiments in mice.

Note that full information on the approval of the study protocol must also be provided in the manuscript.
